# Clinically Isolated Syndrome According to McDonald 2010: Intrathecal IgG Synthesis Still Predictive for Conversion to Multiple Sclerosis

**DOI:** 10.3390/ijms18102061

**Published:** 2017-09-27

**Authors:** Philipp Schwenkenbecher, Anastasia Sarikidi, Lena Bönig, Ulrich Wurster, Paul Bronzlik, Kurt-Wolfram Sühs, Refik Pul, Martin Stangel, Thomas Skripuletz

**Affiliations:** 1 Clinical Neuroimmunology and Neurochemistry, Department of Neurology, Hannover Medical School, 30625 Hannover, Germany; schwenkenbecher.philipp@mh-hannover.de (P.S.); sarikidi.anastasia@mh-hannover.de (A.S.); boenig.lena@mh-hannover.de (L.B.); wurster.ulrich@mh-hannover.de (U.W.); suehs.kurt-wolfram@mh-hannover.de (K.-W.S.); refik.pul@uk-essen.de (R.P.); stangel.martin@mh-hannover.de (M.S.); 2 Department of Diagnostic and Interventional Neuroradiology, Hannover Medical School, 30625 Hannover, Germany; bronzlik.paul@mh-hannover.de; 3 Department of Neurology, University Clinic Essen, 45147 Essen, Germany

**Keywords:** clinically isolated syndrome, multiple sclerosis, OCB, CSF

## Abstract

While the revised McDonald criteria of 2010 allow for the diagnosis of multiple sclerosis (MS) in an earlier stage, there is still a need to identify the risk factors for conversion to MS in patients with clinically isolated syndrome (CIS). Since the latest McDonald criteria were established, the prognostic role of cerebrospinal fluid (CSF) and visual evoked potentials (VEP) in CIS patients is still poorly defined. We conducted a monocentric investigation including patients with CIS in the time from 2010 to 2015. Follow-ups of 120 patients revealed that 42% converted to MS. CIS patients with positive oligoclonal bands (OCB) were more than twice as likely to convert to MS as OCB negative patients (hazard ratio = 2.6). The probability to develop MS was even higher when a quantitative intrathecal IgG synthesis was detected (hazard ratio = 3.8). In patients with OCB, VEP did not add further information concerning the conversion rate to MS. In patients with optic neuritis and negative OCB, a significantly higher rate converted to MS when VEP were delayed. In conclusion, the detection of an intrathecal IgG synthesis increases the conversion probability to MS. Pathological VEP can help to predict the conversion rate to MS in patients with optic neuritis without an intrathecal IgG synthesis.

## 1. Introduction

A clinically isolated syndrome (CIS) is defined as a first episode of neurological symptoms caused by inflammation leading to demyelination in the central nervous system (CNS) [1,2]. Multiple sclerosis (MS), as a chronic inflammatory CNS disease, begins in approximately 85% of patients with such an acute clinical episode [1,2,3]. According to the latest revision of the McDonald criteria for the diagnosis of MS from 2010, patients can be diagnosed with MS after a single clinical episode when cranial magnetic resonance imaging (MRI) detects CNS lesions in typical CNS areas together with an asymptomatic contrast enhancing lesion, thus fulfilling the criteria of dissemination in space and time [4]. However, around 60–70% of patients with a first clinical episode do not fulfill these MS diagnosis criteria [1,5]. Since an early disease modifying immunomodulatory treatment is considered to be beneficial in patients who are at high risk to develop MS, it is essential to identify the predictive factors to anticipate the conversion from CIS to MS [6,7]. CSF inflammatory changes and delayed evoked potentials are characteristic findings in patients with a demyelinating disease [8]. Visual evoked potentials as an easy method to examine a demyelinating disease are considered to have a diagnostic value and can deliver prognostic information on the disease course [9,10,11,12,13]. An intrathecal synthesis of IgG antibodies in the form of oligoclonal bands (OCB) is typical and highly prevalent in MS patients, but can also be observed in CIS patients [14]. In a recent study which applied the Poser criteria to diagnose MS, the presence of OCB has been identified as one of the strongest independent predictors for MS [3]. Another study confirmed the crucial role of OCB for prognosis of CIS patients when applying the McDonald criteria of 2010 [15]. Because the prognostic role of CSF findings in patients with CIS according to the latest McDonald criteria is still poorly defined, we performed a thorough evaluation of CSF parameters. Furthermore, the role of visual evoked potential as an additional prognostic marker was investigated. 

## 2. Results

A follow-up of 120 patients with CIS revealed that 50 patients (42%) converted to MS according to the McDonald criteria of 2010, while 70 patients (58%) were assessed as stable CIS (Table 1). MS was diagnosed due to a second clinical relapse in 28 patients (56% in the MS group), while 22 patients presented new CNS lesions on follow-up MRI. The median conversion time for MS was 11 months (range 2–66). The follow-up time of patients with stable CIS was between 24 and 87 months (median 47 months). 

Patients with the brainstem symptoms and spinal cord symptoms as a first clinical episode converted significantly more frequently to MS during follow-up than showing a stable course (Table 1). The majority of patients diagnosed with optic neuritis as the first clinical episode showed a stable course and only 27% of patients converted to MS. 

### 2.1. CSF Parameters Predict Conversion to Multiple Sclerosis (MS) in Clinically Isolated Syndrome (CIS) Patients

**Figure 1:**
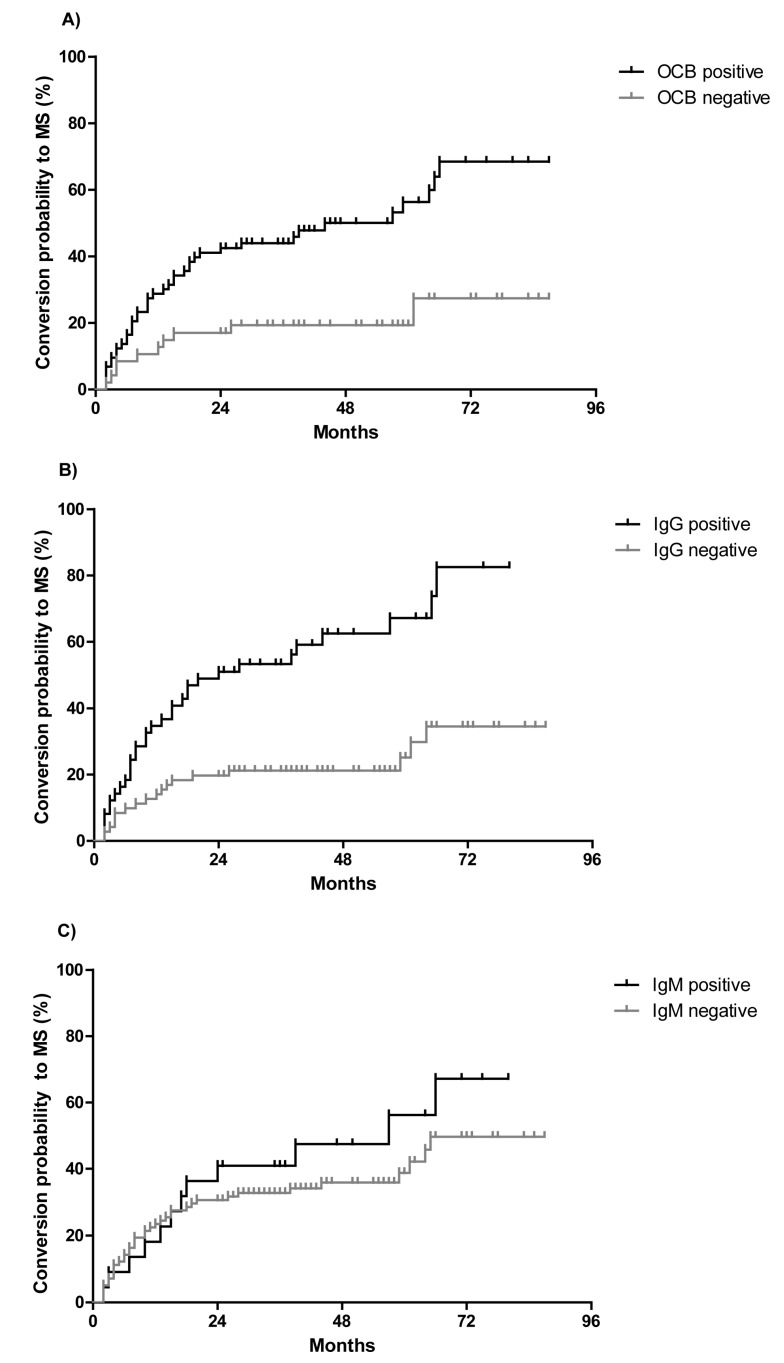
Kaplan-Meier curves for conversation of all clinically isolated syndrome (CIS) patients to multiple sclerosis (MS) in regard to prevalence of OCB restricted to cerebrospinal fluid (CSF) (**A**) intrathecal IgG synthesis; (**B**) and intrathecal IgM synthesis; (**C**) according to the method of Reiber-Felgenhauer.

OCB restricted to CSF were detected in 73 patients (61%) with CIS at baseline examination. During the follow-up the conversion rate to MS was significantly higher in patients with OCB (55%, 40/73 patients) as compared to patients tested negative for OCB (21%, 10/47 patients). CIS patients with positive OCB were more than twice as likely to convert to MS as OCB negative patients (hazard ratio = 2.6, *p* = 0.0005, Figure 1A). The median conversion time to MS was similar in both groups, and thus, not dependent on OCB positivity (11 months in OCB positive patients, 10 months in OCB negative patients; range 2–66 months in both groups). 

A quantitative measured intrathecal synthesis of either IgG, IgM, or IgA according to the method of Reiber-Felgenhauer was present in 50 patients (42%) with CIS at baseline and was always accompanied by OCB positivity. IgG synthesis was found in 48 patients (40%), IgM synthesis in 22 patients (18%), and IgA synthesis in 4 patients (3%). The combination of IgG and IgM was found most frequently in 20 patients (17%), while the combination of IgM and IgA was only found once (1%). Three patients (2%) presented a three-class-reaction of IgG, IgM, and IgA at baseline. 

During the follow-up the conversion rate to MS was significantly higher in patients with intrathecal IgG synthesis (67%, 32/48 patients) as compared to patients without IgG synthesis (33%, 16/48 patients). Patients with the detection of an intrathecal IgG synthesis were more than three and a half times as likely to convert to MS (hazard ratio = 3.8, *p* < 0.0001, Figure 1B). The median conversion time to MS was 11 months in both patients groups, independent if patients exhibited intrathecal IgG synthesis or not. For intrathecal IgM synthesis, a similar trend for the conversion rate to MS failed to be significant (hazard ratio = 1.4, *p* = 0.33, Figure 1C). 12 patients (55%) with intrathecal IgM synthesis converted to MS during follow-up while 10 patients with intrathecal IgM synthesis remained as stable CIS (45%). IgA synthesis occurred in only four patients, and was thus not able to distinguish between groups.

**Figure 2:**
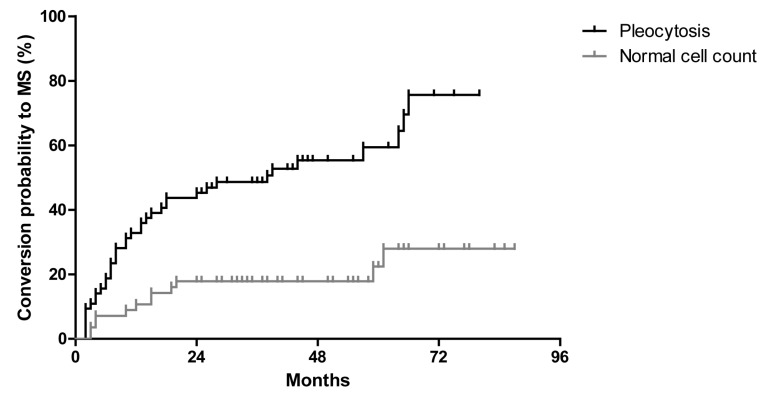
Kaplan-Meier curves for conversation of all CIS patients to MS in regard to prevalence of CSF pleocytosis.

CSF pleocytosis was found in 64 patients with CIS (53%) at the baseline. During follow-up the conversion rate to MS was significantly higher in patients with pleocytosis (59%, 38/64 patients) as compared to patients with normal cell count (41%, 26/64 patients). CIS patients with CSF pleocytosis were three and a half times as likely to convert to MS as patients with normal cell count (hazard ratio = 3.4, *p* < 0.0001, Figure 2). 

The CSF parameters lactate, total protein, and albumin ratio were not able to distinguish between the patients with conversion to MS and stable CIS (Table 2).

### 2.2. OCB Predict the Conversion Rate to MS in CIS Patients Who do not Fulfill MRI Dissemination in Space

**Figure 3:**
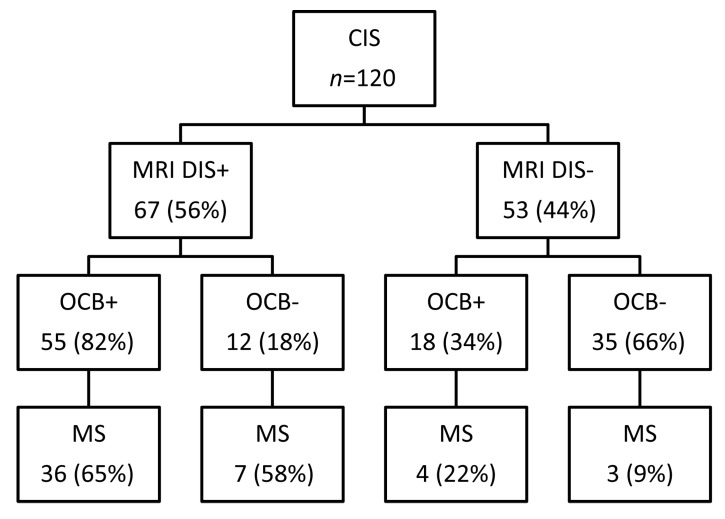
Flow diagram depicting conversion of all CIS patients to MS in regard of the prevalence of OCB restricted to CSF and fulfilling the magnetic resonance imaging (MRI) criteria for dissemination in space.

At baseline examination, 67 patients with CIS (56%) fulfilled the MRI criteria for dissemination in space (Figure 2). The majority of these patients displayed OCB in the CSF (82%). During follow-up, the conversion rate to MS was slightly increased in patients with OCB (65%, 36/55 patients) as compared to patients tested negative for OCB (58%, 7/12 patients, *p* = 0.092, Figure 3) but the result did not reach a significant difference.

In the remaining 53 patients (44%) who did not fulfill the MRI criteria for dissemination in space OCB were found in 18 of them (34%). During the follow-up the conversion rate to MS was significantly higher in patients with OCB (22%, 4/18 patients) as compared to patients tested negative for OCB (9%, 3/35 patients; *p* = 0.018, Figure 3).

### 2.3. The Predictive Role of Visual Evoked Potentials for Conversion of CIS to MS 

**Figure 4:**
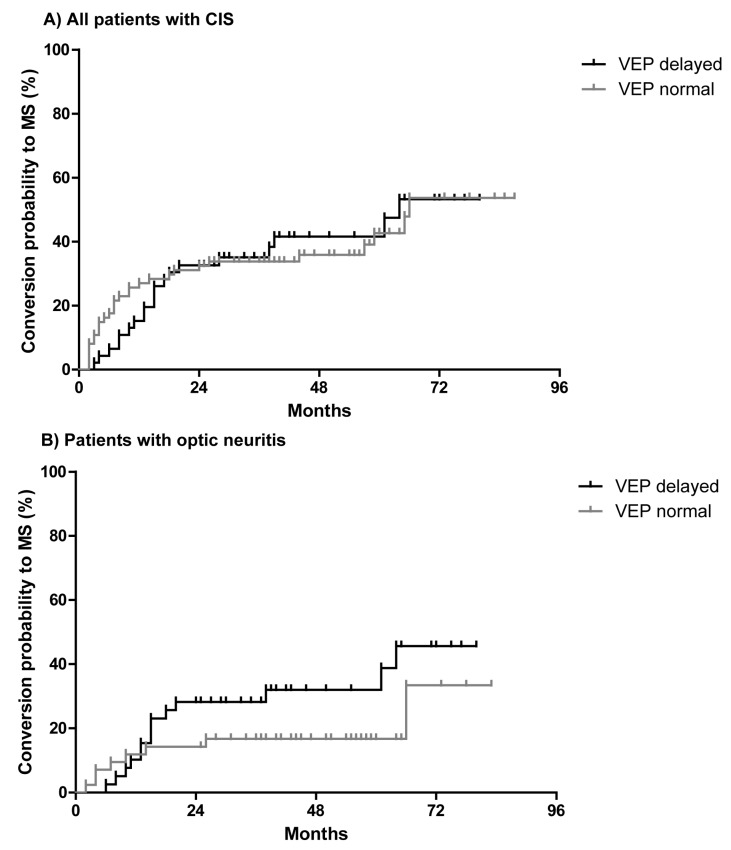
Kaplan-Meier curves for conversation to MS of all CIS patients in regard to normal and delayed visual evoked potentials (VEP) (**A**) and patients with optic neuritis in regard of normal and delayed VEP (**B**).

Electrophysiological examinations including visual evoked potentials (VEP) were performed in all patients with CIS. Examinations revealed delayed VEP indicating a demyelination of the optic nerve in 46 patients (38%) with CIS at the baseline. Delayed VEP at baseline could not discriminate between patients who later converted to MS and patients who remained stable as CIS (hazard ratio = 1.0, *p* = 0.96, Figure 4A). 

When only patients with optic neuritis were analyzed, delayed VEP were found in 49% of them. There was a trend to higher conversion rate in patients with optic neuritis and delayed VEP to develop MS as compared to patients with normal VEP that failed to be significant (hazard ratio = 1.8, *p* = 0.16, Figure 4B).

**Figure 5:**
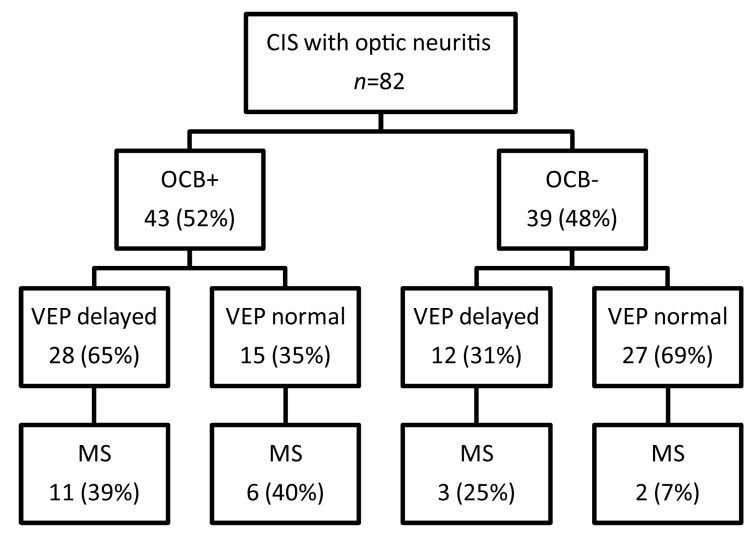
Flow diagram showing conversion of CIS patients with optic neuritis to MS in regard of normal and delayed VEP and the prevalence of OCB.

Further subgroup analyses revealed that patients with optic neuritis and positive OCB in the CSF converted to MS with a similar distribution independently of whether VEP were delayed (39%) or not (40%, Figure 5). However, in patients with optic neuritis and negative OCB, a significantly higher rate converted to MS when VEP were delayed as compared to normal VEP (*p* = 0.008, Figure 5). 

In the group of CIS patients with symptoms other than optic neuritis, delayed VEP were found in six patients who fulfilled the MRI criteria of dissemination in space and converted to MS. Of the six patients with delayed VEP, five were tested OCB positive. In our cohort, delayed VEP were not found in CIS patients with symptoms other than optic neuritis who did not fulfill the MRI criteria of dissemination in space.

## 3. Discussion

Since MS can be diagnosed in an earlier stage according to the last revision of the McDonald criteria in 2010, previously defined risk factors for conversion from CIS to MS need to be reconsidered according to these new guidelines [4]. A recent study has demonstrated that the prevalence of OCB in MS patients persists at a high level [16]. Concomitantly, OCB were less frequently found in patients who did not fulfil the criteria of definitive MS at the first presentation and where thus diagnosed with CIS [16]. Studies applying previous criteria to diagnose MS have shown that the presence of OCB in CIS patients increased the risk to develop MS significantly [3,17,18]. Kuhle and colleagues showed that the presence of OCB in CIS patients duplicated the risk to develop MS independent of the MRI findings, while the detection of MRI lesions in combination with OCB indicated the highest risk [3]. In the present study, CSF parameters in CIS patients diagnosed according to the McDonald criteria of 2010 were analyzed. Our study confirmed the high predictive value of OCB in CSF for conversion to MS. CIS patients with positive OCB were more than twice as likely to convert to MS as OCB negative patients. While the OCB status has been in focus of many studies, further CSF parameters were previously only poorly investigated. We could demonstrate that although a quantitative intrathecal IgG synthesis according to the method of Reiber-Felgenhauer was less frequently detected in patients who converted to MS when compared to OCB as a qualitative method, patients with an increased quantitative IgG production had an even higher risk to develop MS. We could not confirm previous reports that an intrathecal IgM synthesis represents a prognostic factor for conversion of CIS to definitive MS [19,20]. Instead, we found that CSF pleocytosis was associated with a higher risk to convert to MS, which is in contrast to previous reports [3]. However, the previous studies included CIS patients with criteria older than those of McDonald 2010.

Importantly, there are also OCB negative patients who convert to MS. A recent study has shown that MS patients without OCB at the baseline might develop an intrathecal immunoglobulin synthesis in a follow-up CSF investigation [21,22]. In our study, two OCB negative patients who converted to MS received a second lumbar puncture in which OCB in CSF could then be detected. We therefore suggest a follow-up puncture in OCB negative patients within one year in order to confirm the diagnosis and to estimate the prognosis because OCB negative patients have been proposed to have a more benign disease course [14,22]. Alternatively, an investigation of other laboratory predictors in patients with negative OCB might be helpful [23,24,25,26]. A polyspecific response to neurotropic viruses such as measles, rubella, and varicella zoster, with corresponding antibodies in the CSF, which is called “MRZ reaction”, could be found frequently in CIS patients who converted to MS [27]. Another neurotropic virus, the Epstein-Barr virus has been considered as a trigger factor for developing MS and a recent study suggested the detection of an antibody response against the Epstein-Barr virus as a prognostic marker for disease conversion [25]. A unique antibody gene mutation pattern in CSF B-cells and specific molecular changes in circulating CD4^+^ T-Cells were recently identified as risk factors for MS [24,28]. Proteomic analysis of CSF samples is another promising method that suggested chitinase 3-like 1 as a biomarker [23].

Although most studies could not find a significant relationship between clinical presentation at the baseline and the risk to develop MS, some studies suggested that patients with optic neuritis are at a lower risk for conversion to definite MS, which we could confirm [5,29,30,31]. In our cohort, CIS patients with brainstem and spinal cord lesions were at a higher risk, while patients with optic neuritis were at a lower risk to convert to MS. Since the spectrum of diseases leading to visual impairment is broad, clinical examination to objectify patients symptoms are limited and even so considered typical optic neuritis needs to be differentiated from other causes of an acute optic neuropathy [32,33]. Visual pathways lesions due to demyelination can be detected by delayed VEP latencies even in patients without visual symptoms [34,35,36,37]. Therefore, the detection of sub-clinical lesions of the visual pathway has become an important aspect in the assessment of newly diagnosed MS cases [38,39]. While electrophysiological examinations are not a part of the McDonald 2010 criteria, studies demonstrated that VEP latency combined with MRI could improve the accuracy of MS prediction [9,40]. Our results confirm that delayed VEP represent an additional risk factor for conversion to MS in OCB negative CIS patients with optic neuritis. In OCB, positive patients with optic neuritis VEP were not able to discriminate between patients who develop MS or remain stable with CIS. As many patients with CIS present with optic neuritis as first clinical episode, we suggest VEP examination in addition to lumbar puncture to gain additional information to stratify the risk to convert to MS.

In conclusion, although the revised McDonald criteria of 2010 do not consider CSF diagnostics and electrophysiological examination mandatory, the detection of OCB, and delayed VEP can help to identify CIS patients with a high risk to convert to MS.

## 4. Methods

### 4.1. Patients

The study investigated patients with symptoms suggestive for a demyelinating disease who were diagnosed with CIS in the time from 2010 to 2015 at the Department of Neurology of the Hannover Medical School. Patients diagnosed with MS according to the McDonald criteria of 2010 were not included. This group of CIS patients with baseline characteristics was identified in a previous study [16]. In the underlying study, follow-up clinical and MRI parameters were analysed to identify patients who develop MS. The aim of the study was to identify risk factors for developing MS that were evaluated at baseline examination with special interest on CSF parameters and visual evoked potentials. 

Of 189 patients diagnosed with CIS in the time from 2010 to 2015, follow-up data was available for 125 patients (66%). In three patients, an alternative diagnosis for the initial clinical episode appeared during follow-up (keratoconus, keratoectasia, pseudotumor cerebri) and therefore these patients were excluded. Two other patients were not included due to death caused by brain tumor and brain bleeding. 

MS mimicking diseases such as connective tissue diseases and infectious diseases were excluded by laboratory testing (antinuclear antibodies, anti-DNA antibodies, antineutrophil cytoplasmic antibodies, antiphospholipid antibodies, HIV, antibodies to borrelia burgdorferi, antibodies to Treponema pallidum). Baseline clinical data, CSF findings, and MRI of the brain were available for all of the patients. Follow-up data included information about the clinical course with further relapses and MRI results for all patients. 

This investigation was approved by the institutional ethics committee of the Hannover Medical School (No. 7483, 23 May 2017).

### 4.2. CSF and Serum Analytical Procedures

All of the CSF and serum laboratory tests were performed in the neurochemistry laboratory of the Department of Neurology [41,42]. After microscopic cell counting and cell differentiation, CSF samples were centrifuged and total protein (cut-off = 500 mg/L) was determined by a Bradford dye-binding procedure [43]. Immunoglobulin classes IgG, IgA, IgM, and albumin were measured in CSF and serum by kinetic nephelometry (Beckman Coulter IMMAGE). The CSF-serum albumin quotient (QAlb) was used as a marker for the blood-CSF barrier function. The upper normal reference value of the QAlb was calculated by using the formula QAlb = 4 + (age in years/15) [43]. Evidence of an intrathecal synthesis of IgG, IgA, and IgM was based on the calculation according to the method of Reiber-Felgenhauer [43]. CSF-specific OCB were determined by isoelectric focusing in polyacrylamide gels with consecutive silver staining. All of the used CSF analytic methods are quality controlled by participating in the external INSTAND survey program [44].

### 4.3. Magnetic Resonance Imaging (MRI)

All of the patients were examined by cerebral MRI, including a T1-weighted, T2-weighted, and gadolinium-enhancing T1-weighted sequence at baseline. Spinal MRI at the baseline was additionally considered whenever available. The number, location, and gadolinium uptake of CNS lesions was analyzed and classified according to the McDonald Criteria of 2010 (Swanton MRI criteria of 2006) [45]. Evidence of new CNS lesion on follow-up MRI examination with reference to baseline MRI was assessed as dissemination in time.

### 4.4. Visual Evoked Potentials

VEP were performed by trained technicians on a Natus System according to standard methods. The latency of onset and the peak to peak amplitudes of the initial negative peak (N100) and the following positive peak (P100) were measured. An absolute latency of P100 >120 ms or a side difference >10 ms was defined as prolonged VEP [46,47,48,49].

### 4.5. Statistical Analysis

The statistical analysis was performed by GraphPad Prism (La Jolla, CA, USA; version 5.02). Sample values were described as median and range. Statistical significance in categorical data was assessed by Fisher’s exact test while Mann-Whitney *U*-test was performed for two independent samples. Statistical significance was considered for *p*-values <0.05. Values were tested for normal distribution by the D’Agostino-Pearson normality test. Kaplan-Meier surviving analysis was used to estimate probability for conversion to MS.

## Figures and Tables

**Table 1:** Demographic and clinical data of patients with clinically isolated syndrome (CIS) who converted to multiple sclerosis (MS) and patients with stable CIS. *p*-values indicate comparison between MS and patients with stable CIS.

Characteristics	Converted to MS (*n* = 50)	Stable CIS (*n* = 70)	*p*-Value
Age, median (range)	33 (17–73)	35 (16–60)	0.33
Females	33/50 (66%)	45/70 (64%)	0.65
Optic neuritis	22/82 (27%)	60/82 (73%)	0.0001
Paresis/sensory symptoms	4/7 (57%)	3/7 (43%)	0.07
Brainstem symptoms	9/13 (69%)	4/13 (31%)	0.0001
Spinal cord symptoms	15/18 (83%)	3/18 (17%)	0.0001

**Table 2:** Cerebrospinal fluid findings of patients with clinically isolated syndrome (CIS) who converted to multiple sclerosis (MS) and patients with stable CIS. *p*-values indicate comparison between MS and patients with stable CIS.

CSF Findings	Converted to MS (*n* = 50)	Stable CIS (*n* = 70)	*p*-Value
CSF oligoclonal bands	40/50 (80%)	33/70 (47%)	<0.0001
Intrathecal synthesis (Reiber graphs)	32/50 (64%)	18/70 (26%)	<0.0001
IgG	32/50 (64%)	16/70 (23%)	<0.0001
IgM	12/50 (24%)	10/70 (14%)	0.10
IgA	1/50 (2%)	3/70 (4%)	0.68
Pleocytosis (≥5 cells/µl)	38/50 (76%)	26/70 (37%)	<0.0001
Lactate (>3.5 mmol/L)	0 (0%)	0 (0%)	1
Protein (>500 mg/L)	13/50 (26%)	14/70 (20%)	0.31
Blood-CSF-barrier dysfunction	12/50 (24%)	17/70 (24%)	1
